# Transition of Adolescents With Diabetes Mellitus to Adult Care at the Ho Teaching Hospital in Ghana

**DOI:** 10.1155/pedi/7577764

**Published:** 2025-02-05

**Authors:** Ruth Nimota Nukpezah, Cyril Charles Tsigbe

**Affiliations:** ^1^Department of Preventive Health Nursing, University for Development Studies, Tamale, Ghana; ^2^Ghana Health Service, Ho Teaching Hospital, Ho, Ghana

**Keywords:** adolescents, adult care, diabetes mellitus, Ghana, transitioning

## Abstract

**Background:** Transitioning adolescents with diabetes from pediatric to adult care poses significant challenges, especially in low-resource settings like Ghana. Poorly coordinated transitions can disrupt care continuity and adversely impact health outcomes.

**Objective:** This study explored how adolescents with diabetes mellitus (DM) transition from pediatric to adult care at Ho Teaching Hospital, Ghana.

**Methods:** A qualitative exploratory-descriptive design was used. Semistructured interviews were conducted with 15 adolescents and their caregivers. Thematic analysis was applied to identify key themes and subthemes.

**Findings:** Six key themes emerged: (1) inadequate education on DM management, with gaps in adolescents' and caregivers' understanding of the disease and emergency symptoms; (2) limited self-management skills, with caregivers performing most care tasks; (3) poor timing and uncoordinated transfer, with abrupt transitions at age 13; (4) overreliance on caregivers, as caregivers were hesitant to shift responsibilities to adolescents; (5) limited adolescent involvement in care decisions, with healthcare providers engaging more with caregivers; and (6) recommendations for transition improvement, including raising the transfer age, providing skills training, and establishing a transition clinic.

**Conclusion:** The study underscores the need for a structured, developmentally appropriate transition process with targeted education, skills training, and adolescent participation to promote self-management and improve transition outcomes for adolescents with DM.

## 1. Introduction

Globally, ~8000 children develop type 1 diabetes mellitus (DM) annually, with an estimated 50,000 children aged 0–14 years living with the disease Sobota, [1]. In Africa, the International Diabetes Federation reported that as of 2017, 50,600 children and adolescents were living with type 1 diabetes [2]. In Ghana, incidence and prevalence figures remain unclear, but Sarfo-Kantanka et al. [3] estimated the prevalence to be between 52.8 and 73.0 per 100,000 children. Advances in understanding, diagnosis, and managing chronic childhood illnesses have extended the lifespan of children with long-term conditions, allowing many to live well into adulthood [4]. Consequently, hundreds of young people with chronic conditions, including diabetes, are expected to enter a transition phase where they must gradually assume responsibility for their care [5, 6].

Transitioning from pediatric to adult care poses unique challenges due to differences in approaches to care, provider-client interactions, and follow-up procedures. Adolescents face abandonment, role confusion, and unpreparedness during the transition to adult care [7–9], worsened by poor provider coordination [10] and chronic conditions in resource-limited settings [11]. These differences necessitate a coordinated effort to ensure a seamless transition. Moreso, they face unstructured transition processes [7–9, 12]. Structured clinics and guidelines can address these gaps [12–14], while resource-limited settings face added burdens from conditions like diabetes and HIV [11, 15, 16]. The absence of a coordinated process often results in an abrupt transfer of adolescents to adult care providers, leading to a 2.5-fold increase in the odds of poor glycemic control. Reviews of intervention studies have shown that structured transition programs can effectively reduce adverse outcomes associated with moving from pediatric to adult care Sobota [1]. The transition period has become a key focus in pediatric and adult medicine, requiring considerable resources to ensure success. Poor transition planning is linked to lower attendance at adult clinics and increased short- and long-term complications, including hospitalizations and deaths from diabetic ketoacidosis [17].

Despite pediatric and adult diabetes clinics in Ghana, there is limited data on transition processes to support adolescents with diabetes in becoming self-sufficient. The study area reflects the broader national picture, where no scientific data exists on how children with diabetes and other chronic diseases transition to adult care. While anecdotal evidence suggests that transitioning children with diabetes and other chronic conditions to adult care is highly challenging, scientific data to inform best practices are lacking. This study addresses this gap by examining current transition practices, exploring client experiences, and gathering suggestions to develop a system that better supports clients and healthcare teams.

### 1.1. Aim

The study explored how adolescents with DM transition from pediatric care to adult care at the Ho Teaching Hospital in Ghana.

## 2. Methods

### 2.1. Design

A qualitative exploratory-descriptive design was employed to identify and describe how adolescents with DM transition from pediatric care to adult care. Thematic analysis was used to analyze the data, emphasizing identifying, analyzing, and interpreting qualitative data patterns to identify common themes and insights [18, 19]. This method provided a flexible approach for extracting insights and drawing conclusions from the data.

### 2.2. Participants and Sampling

Participants were recruited from the pediatric and adult DM clinics at the Ho Teaching Hospital. The participants included adolescents with DM and their caregivers. The clinic register and a one-on-one approach identified adolescents (diagnosed at least 6 months before the study) and their caregivers seeking care at the hospital. Adolescents diagnosed with DM less than 6 months before the study were excluded to ensure that participants had sufficient experience with the condition and healthcare system.

A nonprobability purposive sampling method was used, with a sample size of 15 participants. This sample size was guided by the need for in-depth exploration and data saturation, which the 13th respondent reached. Two additional interviews were conducted to confirm saturation. Fourteen participants were below 18 years of age and were interviewed with their caregivers, while one participant, aged 18, was interviewed alone. The adolescents' ages ranged from 10 to 18 years, with disease durations between 1 and 8 years.

### 2.3. Data Collection

Data collection occurred from April to June 2023. Semistructured interviews were conducted with adolescents and their caregivers to obtain in-depth information. Interviews were held in a private room within the hospital, each lasting 40–50 min. A semistructured interview guide adapted from previous research was used, and all sessions were audio recorded. Interview questions focused on participants' knowledge of DM, self-care practices, and experiences with the transition process.

The interviewer explained the study's purpose, facilitated the conversation, and ensured participants' responses were clear and comprehensive. To maintain consistency, all interviews followed the same procedure, including using the same interview guide. Probes and follow-up questions were used to obtain further clarification and deeper understanding. After each session, participant responses were summarized to ensure accuracy.

### 2.4. Interviewer's Characteristics

The interviews were conducted by Ruth Nimota Nukpezah, a student with extensive training in qualitative research methods, and Cyril Charles Tsigbe, an experienced qualitative researcher who has published multiple peer-reviewed articles using qualitative methodologies. Ruth's expertise includes the use of phenomenology and exploratory-descriptive designs, particularly in healthcare research. Their combined experience ensured the application of rigorous qualitative research techniques, including effective probing, clarification, and active listening, facilitating the collection of rich, in-depth data. Both interviewers maintained reflexivity throughout the process to minimize bias, enhance credibility, and ensure that participants' voices were authentically represented.

### 2.5. Data Analysis

Thematic analysis was used to analyze the data concurrently with data collection. Audio-recorded interviews were transcribed verbatim, and transcriptions were cross-checked against recordings to ensure accuracy. All transcripts were reviewed multiple times for familiarization. Codes were assigned to recurring ideas, grouped into subthemes, and then collated to form the main themes. Thematic analysis enabled the identification of patterns in participants' experiences and perspectives. Both contributors analyzed the responses independently to ensure inter-rater reliability, with similar themes emerging across both analyses. Questions guiding the analysis included “What patterns are seen in the data?” and “What commonalities exist across participant responses?"

### 2.6. Validity and Reliability

Descriptive validity was achieved by ensuring participant responses were captured accurately through verbatim transcription and paraphrasing during interviews. Theoretical validity was ensured through discussions with colleagues and alignment with the study's theoretical framework. Credibility was established by recruiting participants who met the inclusion criteria, ensuring confidentiality, and using pseudonyms to protect identities. Using a consistent interview guide, standardized data collection procedures, and a detailed audit trail supported the reliability of the study.

### 2.7. Ethical Considerations

Ethical approval was obtained from the Human Research, Publication, and Ethics Committee of the School of Medical Sciences of Kwame Nkrumah University of Science and Technology (reference number CHRPE/AP/248/23). Permission was also obtained from the management of the Ho Teaching Hospital to recruit participants and collect data at the facility.

Participants were briefed on the purpose of the study, and their consent was obtained before participation. Caregivers provided written informed consent for minors, while adolescents aged 18 provided written consent. For nonliterate participants, information was explained in Ewe, with the support of a literate interpreter who confirmed the accuracy of the information provided. Participants were informed that their participation was voluntary and that they could withdraw at any point without repercussions. Data confidentiality was maintained by collecting information in a private room and using pseudonyms to ensure anonymity.

### 2.8. Data Saturation

Data saturation was reached after the 13th participant, as no new information was obtained. Two additional participants were interviewed to confirm saturation.

### 2.9. Interview Guide

#### 2.9.1. Opening Questions

1. Can you tell me about your child, including their age, education level, time of first diagnosis, and about yourself, such as your ethnicity, education level, and religion?

#### 2.9.2. Key Questions

1. Can you describe your understanding of diabetes, including its symptoms, causes, and signs that prompt the need for urgent care?
2. How is diabetes managed in your household, and what role does your child play in managing their care?

Probe: What challenges have you faced in encouraging your child to take on aspects of their care?

3. What education or training have you and your child received from healthcare providers regarding diabetes management?

Probe: How effective was this education, and what aspects were most beneficial?

4. Can you share your experience with your child's transition to the adult clinic?

Probe: How were you informed and prepared for the move, and what support did you receive from healthcare providers?

5. What changes or improvements would you recommend to make the transition process smoother and more effective for children with diabetes at this facility?

Probe: Are there specific resources or support mechanisms that would have been helpful to you and your child?

## 3. Findings

### 3.1. Demographics of Participants

Table 1 provides an overview of participant demographics, including the adolescents' ages (ranging from 10 to 18 years), their current education levels, and the years since diagnosis (ranging from 1 to 8 years). Additionally, it highlights the primary caregivers' education levels and their role in providing information for the study.

The study data analysis revealed six key themes with associated subthemes that highlight the transition experiences of adolescents with DM from pediatric to adult care at the Ho Teaching Hospital.

## 4. Main Findings

### 4.1. Inadequate Education on Diabetes Management

Health education on diabetes, a crucial pillar of the transition process, was found to be inadequate. Adolescents and caregivers lacked comprehensive knowledge of the disease, its causes, and symptoms. The absence of structured education sessions by healthcare providers contributed to this gap.

Participants highlighted this challenge. For instance, a 10-year-old participant stated, “They told me that I have diabetes, which means that there is too much sugar in my blood…” (Resp. 2, 10 years). Another participant noted, “When I feel easily tired, I start sweating or my body starts to shake” (Resp. 3, 12 years), indicating limited awareness of emergency symptoms. Caregivers echoed similar sentiments, with one parent mentioning, “The doctor only explained it at diagnosis but never taught us the symptoms to watch for” (Resp. 14, caregiver of a 10-year old). These statements align with prior literature that emphasizes the role of education in promoting effective healthcare transitions [20].

#### 4.1.1. Subthemes

• Limited understanding of DM: Adolescents and caregivers displayed shallow knowledge of the disease, often limited to their own experiences.
• Inadequate knowledge of emergency signs: Participants could not identify emergency symptoms, relying on caregivers to recognize signs such as tiredness or sweating.

### 4.2. Limited Self-Management Skills

Transitioning from pediatric to adult care requires adolescents to acquire self-management skills. However, many participants revealed underdeveloped self-management skills, as parents performed most caregiving tasks. Adolescents often struggle with essential skills like monitoring glycemic levels or administering insulin injections.

Several participants provided insight into this challenge. For example, one caregiver remarked, “We tried to teach him the injection, but he was having some difficulties” (Resp. 4, caregiver of a 13-year old). Similarly, another participant stated, “He is still too young to understand anything to be able to handle any of those things, so we take care of every aspect of the care” (Resp. 14, caregiver of a 10-year old). Adolescents also expressed concerns, with one saying, “I know how to check my sugar, but I don't know what to do if it's too high” (Resp. 12-, 10-year old).

#### 4.2.1. Subthemes

• Caregiver reliance: Caregivers took responsibility for most aspects of care, as evidenced by statements such as, “We don't even allow her older siblings to handle anything” (Resp. 12, caregiver of a 10-year old).
• Poor self-care competency: Adolescents had difficulty understanding glycemic readings, and caregivers believed that their children were too young to manage care independently.

### 4.3. Poor Timing and Uncoordinated Transfer to Adult Clinic

The Ho Teaching Hospital follows a policy of transferring adolescents to adult care at 13. However, many caregivers and adolescents found this process abrupt and inadequately managed. Most participants reported receiving little to no information about the transfer, leaving them unprepared.

Caregivers expressed dissatisfaction with the process. For instance, one caregiver stated, “We just came to the clinic 1 day and were told that we should go to the adult side because she is now 13 years old” (Resp. 2, caregiver of a 13-year old). Another parent mentioned, “I heard from another parent that when they turn 13, they move to adult care” (Resp. 9, caregiver of a 13-year old). This sudden transfer without adequate preparation left adolescents and caregivers confused and unprepared for the demands of adult care.

#### 4.3.1. Subthemes

• Lack of communication on transfer process: Some caregivers were informed about the transfer by other parents rather than clinicians.
• Unprepared movement to adult care: Caregivers and adolescents felt unprepared for the transition due to the absence of a structured plan.

### 4.4. Role of Caregivers in Transition and Care Management

Caregivers are critical in supporting adolescents during the transition to adult care. However, their involvement was often marked by overprotection, with caregivers reluctant to relinquish control of care responsibilities.

Caregivers shared their experiences, with one parent noting, “We don't even allow her older siblings to handle anything” (Resp. 12, caregiver of a 10-year old). Another caregiver expressed a similar sentiment, stating, “At this point, I think he is still too young to do those things, so I prefer to do them for him now” (Resp. 14, caregiver of a 10-year old). These findings are consistent with Lapp [21], who identified overprotection as a barrier to effective transition planning.

#### 4.4.1. Subthemes

• Over-involvement of caregivers: Parents managed most care-related activities, as seen in the response, “We don't even allow her older siblings to handle anything” (Resp. 12).
• Lack of caregiver confidence in adolescent ability: Caregivers expressed doubts about the readiness of adolescents to assume care responsibilities.

### 4.5. Limited Involvement of Adolescents in Care Decisions

Participants revealed that healthcare providers rarely engaged adolescents in care decisions, often directing information and guidance to the caregivers. While some adolescents noted limited involvement, others desired more participation.

For example, one adolescent noted, “They try to make me understand, so sometimes they talk to me like a small child” (Resp. 8-, 14-year old). Another adolescent said, “They talk to my parents, but I also want to know what's happening” (Resp. 12-, 10-year old). These responses indicate that adolescents want to be more involved in discussions about their care and the transition process.

#### 4.5.1. Subthemes

• Minimal adolescent participation in care planning: Adolescents indicated that clinicians spoke mainly to their parents.
• Desire for more involvement: Adolescents desire to be more engaged in discussions about their care and transition process.

### 4.6. Suggestions for Improving the Transition Process

Caregivers and adolescents provided several suggestions for improving the transition process. Key recommendations included revising the age of transfer, enhancing the role of clinicians in preparing families for transition, and establishing a dedicated transition clinic for adolescents.

One caregiver stated, “I would suggest that they can be seen at the children's clinic until around 18 years” (Resp. 9, caregiver of a 13-year old). Another suggestion was the creation of a dedicated adolescent transition clinic, with one caregiver stating, “Or they can have a clinic for young people separately from the adult clinic, where they can help us teach them certain things before mixing them with adults” (Resp. 14, caregiver of a 10-year old). These suggestions align with best practices in transitional care and highlight the need for a developmentally appropriate approach.

#### 4.6.1. Subthemes

• Age of transfer: Caregivers suggested delaying the age of transition from 13 to 18.
• Skills training for adolescents: Caregivers proposed that clinicians incorporate skill training for self-care, such as checking glycemic levels and understanding diabetes management.
• Creation of a transition clinic: Caregivers recommended establishing a transition clinic tailored to the needs of adolescents, separate from the adult clinic.

The findings highlight several gaps in the transition process for adolescents with diabetes at the Ho Teaching Hospital. Key challenges include inadequate education on diabetes, limited acquisition of self-management skills, and poor preparation for the transfer to adult care. The overreliance on caregivers and the limited engagement of adolescents in care decisions further complicate the transition process. Participants proposed several recommendations, including increasing the transfer age, providing skills training, and establishing an adolescent transition clinic. Addressing these gaps could facilitate smoother transitions and better health outcomes for diabetic adolescents.

## 5. Discussion

The findings reveal that education on diabetes management, a fundamental component for successful healthcare transitions, is significantly lacking at the Ho Teaching Hospital. Comprehensive disease-specific education promotes self-management and prepares adolescents for independent care [22]. However, participants in this study reported insufficient formal education on diabetes and the transition process. Adolescents and caregivers relied primarily on informal sources and fragmented information during clinical encounters. Participants' responses suggested that this knowledge gap led to heightened anxiety, uncertainty, and a lack of readiness for the transition process. Adolescents with minimal understanding of diabetes and its management were less equipped to participate in their care, a finding supported by Varty et al. [23]. The lack of a formalized education strategy within the study site underscores the urgent need for a structured, purposeful education program to support adolescents, and their caregivers in understanding the disease, self-management, and the expectations of adult care.

Transitioning from pediatric to adult care requires adolescents to acquire essential self-management skills. The consensus statements on healthcare transition emphasize the need for a clear and deliberate plan to develop self-management capabilities [2, 5]. However, this study revealed that adolescents lacked vital self-care skills, such as checking glycemic levels, managing medications, and understanding dietary requirements. Participants indicated that caregivers, not healthcare providers, were responsible for teaching self-care skills, a role that many caregivers felt ill-prepared to assume. Kamau et al. [2] found that only 16% of adolescents in similar studies had received skill training from clinicians. This finding is mirrored in this study, where caregivers equipped adolescents with skills to manage their care independently. The absence of clinician-guided training programs limits adolescents' confidence and readiness to manage their care independently. Meleis's transitions theory underscores the importance of identifying milestones, which is often achieved through structured skills training. However, findings from this study suggest that this critical aspect is largely unmet at the Ho Teaching Hospital, highlighting the need for a structured training curriculum for adolescents.

Active participation of adolescents in care decisions is a core principle of successful healthcare transition. However, this study revealed that adolescents were often passive participants in discussions about their care. Clinicians focused on caregivers, and adolescents felt excluded from key decisions, as evidenced by one participant's statement, “They try to make me understand, so sometimes they talk to me like a small child” (Resp. 8). Limited engagement in decision-making reduces adolescents' autonomy and self-efficacy. Studies have shown that when adolescents actively participate in their care, they develop confidence in self-management and are better equipped to navigate adult healthcare settings [23]. Incorporating adolescents into care discussions empowers them to understand and take responsibility for their condition, an essential milestone for a successful healthcare transition.

The Ho Teaching Hospital's policy of transferring adolescents to adult care at age 13, regardless of readiness, was a major source of concern for participants. Most caregivers and adolescents described this transition as abrupt, without formal preparation or support. They were “informed to report to the adult clinic on their next visit” (Resp. 2). This approach runs counter to global best practices, which advocate for a gradual, developmentally appropriate transition process [5, 6]. Participants suggested that the transition age should be raised to 18, aligning with readiness-based rather than age-based transitions. This perspective is consistent with Ödling et al. [24], who argued that the transition should be based on developmental milestones, not just age. The abrupt transfer at the Ho Teaching Hospital mirrors findings from sub-Saharan Africa, where similar transfer models have been linked to poor health outcomes [25, 26]. Structured and phased transition programs that include preparation, education, and progressive involvement of the adolescent in their care have been shown to improve health outcomes [27].

Caregivers play a critical role in supporting adolescents during the transition process. However, this study revealed that caregivers often maintained complete control over the adolescents' care, leaving little room for adolescents to practice self-care. This over-reliance on caregivers was driven by a lack of confidence in adolescents' ability to manage their care. For example, one caregiver stated, “He is too young to handle this on his own, so I prefer to do it for him” (Resp. 14). Overprotective caregiving behaviors, as discussed by Lapp [21], have been shown to hinder the development of self-care skills in adolescents. This study supported this finding, where caregivers' reluctance to delegate care responsibilities resulted in adolescents' limited ability to take on key aspects of diabetes management. To address this issue, caregivers must be supported in gradually transferring care responsibilities to adolescents. Clinicians should facilitate this process by providing guidance and support to caregivers and fostering adolescents' confidence to take on greater responsibility.

Caregivers and adolescents offered suggestions for improving the transition process at the Ho Teaching Hospital. Key recommendations included increasing the transition age, incorporating skills training, and establishing a dedicated adolescent transition clinic. The suggestion to raise the transfer age from 13 to 18 is consistent with international best practices, which advocate for a developmental approach to transition. This approach considers adolescents' cognitive maturity, as seen in reports from the Quality Care Commission [20] and studies by Ödling et al. [24]. Participants also recommended that healthcare providers be more proactive in training adolescents to manage self-care tasks. This approach aligns with the milestone-based transitions discussed by Meleis's transitions theory. By ensuring that adolescents meet specific developmental milestones before transfer, healthcare providers can better prepare them for adult care. Another key suggestion was to establish a dedicated adolescent transition clinic. This clinic would provide youth-friendly services that address the unique needs of adolescents. Similar models, such as those advocated by Foster et al. [28] and Dahourou et al. [29], have increased retention in care and improved health outcomes for adolescents with chronic conditions. Such a facility would provide an environment for skills training, caregiver support, and gradual introduction to adult care processes, which aligns with the findings of this study.

## 6. Limitations

The study's small sample size of 15 participants, limited to a single hospital, may not reflect broader experiences. Excluding healthcare providers' perspectives further narrowed the scope, focusing solely on adolescents and caregivers. The cross-sectional design captured only a snapshot of experiences, omitting the dynamic nature of transition over time. Additionally, the interview guide, though adapted, may not have fully addressed culturally specific factors unique to the Ghanaian context. These constraints affect the transferability and applicability of the findings beyond the study site.

## 7. Conclusion

The findings of this study reveal significant gaps in the transition process for adolescents with diabetes at the Ho Teaching Hospital. Critical areas for improvement include enhanced education on diabetes management, skills training for self-care, better adolescent involvement in care decisions, and a structured, gradual transition process. The abrupt transition at age 13 is not aligned with best practices, and caregivers play an overly dominant role in the process.

Participants proposed several solutions, including raising the transition age to 18, implementing structured skills training programs, and establishing a dedicated adolescent transition clinic. These interventions would support the smooth transition of adolescents into adult care and enhance self-management capabilities. The study's findings underscore the need for a structured and holistic approach to transition, as supported by global best practices and the principles of Meleis's transitions theory. Addressing these issues would reduce caregiver burden, promote adolescent independence, and ultimately improve health outcomes for adolescents with diabetes.

## Figures and Tables

**Table 1:** Demographics of participants.

Part. no.	1	2	3	4	5	6	7	8	9	10	11	12	13	14	15
*Pseudonym*	*Resp. 1*	*Resp. 2*	*Resp. 3*	*Resp. 4*	*Resp. 5*	*Resp. 6*	*Resp. 7*	*Resp. 8*	*Resp. 9*	*Resp. 10*	*Resp. 11*	*Resp. 12*	*Resp. 13*	*Resp. 14*	*Resp. 15*
Age (years) of adolescent	12	10	12	13	13	18	10	14	13	12	11	10	12	10	11
Curent educ. level of adolescent	Prim 6	Prim 4	JHS 1	Prim 6	JHS 1	SHS 2	Prim 5	JHS 1	Prim 6	JHS 1	Prim 5	Prim 5	Prim 5	Prim 4	Prim 4
Years since diagnosis	3	2	2	2	4	8	1	5	3	3	2	3	1.5	2	2
PCG education level	SHS	Tert	Tert	Tert	SHS	N/A	JHS	Tert	Tert	Prim	Tert	SHS	JHS	Prim	SHS
PCG religion	Chris	Chris	Chris	Chris	Chris	N/A	Chris	Chris	Chris	Chris	Chris	Chris	Chris	Chris	Chris
PCG ethnicity	Ewe	Ewe	Akan	Ewe	Ewe	N/A	Guan	Ewe	Ewe	Ewe	Akan	Ewe	Ewe	Ewe	Guan
Source of info (adol, PCG, or both)	Both	Both	Both	Both	Both	Adol	Both	Both	Both	Both	Both	Both	Both	Both	Both

## Data Availability

Generated transcripts from which data were analyzed are kept anonymous, and an extract from them could be made available upon request from the corresponding author.
